# Supplementary material for: Individual- and group-level network-building interventions to address social isolation and loneliness: A scoping review with implications for COVID19
Source: PLoS One. 2021 Jun 25;16(6):e0253734. doi: 10.1371/journal.pone.0253734 (PMC8232435; doi:10.1371/journal.pone.0253734)
Supplement: S1 Appendix — (DOCX) [file pone.0253734.s001.docx]

**S1 Appendix: SCOPUS search strategy**

TITLE-ABS(

(

(network* W/2 intervention*) OR

(network* W/2 experiment) OR

(network* W/2 weav*) OR

(network* W/2 rewir*) OR

(network* W/2 build*) OR

(network* W/2 outcome*) OR

(harness* W/2 network*) OR

(alteration W/2 network*) OR

(altering W/2 network*) OR

(strengthen* W/2 network*) OR

(improve* W/3 network*)

) AND (

(

("social network") OR

(network* W/2 analysis) OR

(network* W/2 analyz*)

) AND

(

intervention OR

experiment OR

initiative OR

trial

)

)

)
