# Supplementary material for: Individual- and group-level network-building interventions to address social isolation and loneliness: A scoping review with implications for COVID19
Source: PLoS One. 2021 Jun 25;16(6):e0253734. doi: 10.1371/journal.pone.0253734 (PMC8232435; doi:10.1371/journal.pone.0253734)
Supplement: Attachment — Submitted filename: PONE-D-20-26180_reviewer.pdf [file pone.0253734.s002.pdf]

PLOS ONE

Individual- and Group-Level Network-Building Interventions to Address Social Isolation and Loneliness; A Scoping Review with implications for COVID19

--Manuscript Draft--

**Manuscript Number:**	PONE-D-20-26180
**Article Type:**	Research Article
**Full Title:**	Individual- and Group-Level Network-Building Interventions to Address Social Isolation and Loneliness; A Scoping Review with implications for COVID19
**Short Title:**	Network-Building Interventions to Address Social Isolation and Loneliness
**Corresponding Author:**	Reza Yousefi Nooraie<br>University of Rochester<br>Rochester, NY UNITED STATES
**Keywords:**	Social isolation; loneliness; social network analysis; network interventions
**Abstract:**	Purpose<br><br>Worldwide mandates for social distancing and home-quarantine have contributed to loneliness and social isolation. We conducted a systematic scoping review to identify network-building interventions that address loneliness and isolation, describe their components and impact on network structure, and consider their application in the wake of COVID19.<br><br>Methods<br><br>We performed forward and backward citation tracking of three seminal publications on network interventions and Bibliographic search of Web of Science and SCOPUS. We developed data charting tables and extracted and synthesized the characteristics of included studies, using an iteratively updating form.<br><br>Findings<br><br>From 3390 retrieved titles and abstracts, we included 8 studies. These interventions focused on building networks at either individual- or group-levels. Key elements that were incorporated in the interventions at varying degrees included (a) creating opportunities to build networks; (b) improving social skills; (c) assessing network diagnostics (i.e. using network data or information to inform network strategies); (d) promoting engagement with influential actors; and (e) a process for goal-setting and feedback. The effect of interventions on network structures, or the moderating effect of structure on the intervention effectiveness was rarely assessed.<br><br>Conclusions<br><br>As many natural face-to-face opportunities for social connection are limited due to COVID19, groups already at risk for social isolation and loneliness are disproportionately impacted. Network-building interventions include multiple components that address both the structure of individuals' networks, and their skills and motivation for activating them. These intervention elements could be adapted for delivery via on-line platforms, and implemented by trained facilitators or novice volunteers, although more rigorous testing is needed.
**Order of Authors:**	Reza Yousefi Nooraie
	Keith Warren
	Lisa A. Juckett
	Qiuchang A. Cao
	Alicia C. Bunger
	Michele A. Patak-Pietrafesa

Powered by Editorial Manager® and ProduXion Manager® from Aries Systems Corporation

Additional Information:	
**Question**	**Response**
**Financial Disclosure**<br><br>Enter a financial disclosure statement that describes the sources of funding for the work included in this submission. Review the submission guidelines for detailed requirements. View published research articles from *PLOS ONE* for specific examples.<br><br>This statement is required for submission and **will appear in the published article** if the submission is accepted. Please make sure it is accurate.<br><br>**Unfunded studies**<br>Enter: *The author(s) received no specific funding for this work.*<br><br>**Funded studies**<br>Enter a statement with the following details:<br>• Initials of the authors who received each award<br>• Grant numbers awarded to each author<br>• The full name of each funder<br>• URL of each funder website<br>• Did the sponsors or funders play any role in the study design, data collection and analysis, decision to publish, or preparation of the manuscript?<br>• **NO** - Include this sentence at the end of your statement: *The funders had no role in study design, data collection and analysis, decision to publish, or preparation of the manuscript.*<br>• **YES** - Specify the role(s) played.<br><br>* typeset	The author(s) received no specific funding for this work.
**Competing Interests**<br><br>Use the instructions below to enter a competing interest statement for this submission. On behalf of all authors, disclose any competing interests that could be perceived to bias this work—acknowledging all financial support and any other relevant financial or non-financial competing interests.	The authors have declared that no competing interests exist.

Powered by Editorial Manager® and ProduXion Manager® from Aries Systems Corporation

This statement **will appear in the published article** if the submission is accepted. Please make sure it is accurate. View published research articles from *PLOS ONE* for specific examples.<br><br>**NO authors have competing interests**<br><br>Enter: *The authors have declared that no competing interests exist.*<br><br>**Authors with competing interests**<br><br>Enter competing interest details beginning with this statement:<br><br>*I have read the journal's policy and the authors of this manuscript have the following competing interests: [insert competing interests here]*<br><br>* typeset	
**Ethics Statement**<br><br>Enter an ethics statement for this submission. This statement is required if the study involved:<br><br>• Human participants<br>• Human specimens or tissue<br>• Vertebrate animals or cephalopods<br>• Vertebrate embryos or tissues<br>• Field research<br><br>Write "N/A" if the submission does not require an ethics statement.<br><br>General guidance is provided below. Consult the submission guidelines for detailed instructions. **Make sure that all information entered here is included in the Methods section of the manuscript.**	N/A

**Format for specific study types**<br><br>**Human Subject Research (involving human participants and/or tissue)**<br>• Give the name of the institutional review board or ethics committee that approved the study<br>• Include the approval number and/or a statement indicating approval of this research<br>• Indicate the form of consent obtained (written/oral) or the reason that consent was not obtained (e.g. the data were analyzed anonymously)<br><br>**Animal Research (involving vertebrate animals, embryos or tissues)**<br>• Provide the name of the Institutional Animal Care and Use Committee (IACUC) or other relevant ethics board that reviewed the study protocol, and indicate whether they approved this research or granted a formal waiver of ethical approval<br>• Include an approval number if one was obtained<br>• If the study involved *non-human primates*, add *additional details* about animal welfare and steps taken to ameliorate suffering<br>• If anesthesia, euthanasia, or any kind of animal sacrifice is part of the study, include briefly which substances and/or methods were applied<br><br>**Field Research**<br><br>Include the following details if this study involves the collection of plant, animal, or other materials from a natural setting:<br>• Field permit number<br>• Name of the institution or relevant body that granted permission	
**Data Availability**<br><br>Authors are required to make all data underlying the findings described fully available, without restriction, and from the time of publication. PLOS allows rare exceptions to address legal and ethical concerns. See the PLOS Data Policy and FAQ for detailed information.	Yes - all data are fully available without restriction

A Data Availability Statement describing where the data can be found is required at submission. Your answers to this question constitute the Data Availability Statement and **will be published in the article**, if accepted.<br><br>**Important:** Stating 'data available on request from the author' is not sufficient. If your data are only available upon request, select 'No' for the first question and explain your exceptional situation in the text box.<br><br>Do the authors confirm that all data underlying the findings described in their manuscript are fully available without restriction?	
**Describe where the data may be found in full sentences. If you are copying our sample text, replace any instances of XXX with the appropriate details.**<br><br>• If the data are **held or will be held in a public repository**, include URLs, accession numbers or DOIs. If this information will only be available after acceptance, indicate this by ticking the box below. For example: *All XXX files are available from the XXX database (accession number(s) XXX, XXX.).*<br>• If the data are all contained **within the manuscript and/or Supporting Information files**, enter the following: *All relevant data are within the manuscript and its Supporting Information files.*<br>• If neither of these applies but you are able to provide **details of access elsewhere**, with or without limitations, please do so. For example:<br><br>*Data cannot be shared publicly because of [XXX]. Data are available from the XXX Institutional Data Access / Ethics Committee (contact via XXX) for researchers who meet the criteria for access to confidential data.*<br><br>*The data underlying the results presented in the study are available from (include the name of the third party*	All relevant data are within the manuscript and its Supporting Information files.

and contact information or URL).<br>• This text is appropriate if the data are owned by a third party and authors do not have permission to share the data.<br><br>* typeset	
Additional data availability information:	

Powered by Editorial Manager® and ProduXion Manager® from Aries Systems Corporation

Click here to access/download;Manuscript;manuscript 28july2020.docx

Title: Individual- and Group-Level Network-Building Interventions to Address Social Isolation and Loneliness; A Scoping Review with implications for COVID19

Reza Yousefi Nooraie, PhD, MD. (Corresponding author)
Department of Public Health Sciences
University of Rochester
265 Crittenden Blvd., Rochester, New York 14642
Tel: +1 (585)-485-8015
Reza_Yousefi-nooraie@URMC.Rochester.edu

Keith Warren, PhD
College of Social Work, The Ohio State University
warren.193@osu.edu

Lisa A. Juckett, PhD, OTR/L, CHT
School of Health and Rehabilitation Sciences, The Ohio State University
Lisa.Juckett@osumc.edu

Qiuchang Cao, MSW, PhD Candidate
College of Social Work, The Ohio State University
cao.847@osu.edu

Alicia C. Bunger, MSW, PhD
College of Social Work, The Ohio State University
bunger.5@osu.edu

Michele Patak-Pietrafesa, MSW, ABD, LISW-S
College of Social Work, The Ohio State University
patak-pietrafesa.1@buckeyemail.osu.edu

Wordcount: 4436

ABSTRACT

Purpose: Worldwide mandates for social distancing and home-quarantine have contributed to loneliness and social isolation. We conducted a systematic scoping review to identify network-building interventions that address loneliness and isolation, describe their components and impact on network structure, and consider their application in the wake of COVID19.

Methods: We performed forward and backward citation tracking of three seminal publications on network interventions and Bibliographic search of Web of Science and SCOPUS. We developed data charting tables and extracted and synthesized the characteristics of included studies, using an iteratively updating form.

Findings: From 3390 retrieved titles and abstracts, we included 8 studies. These interventions focused on building networks at either individual- or group-levels. Key elements that were incorporated in the interventions at varying degrees included (a) creating opportunities to build networks; (b) improving social skills; (c) assessing network diagnostics (i.e. using network data or information to inform network strategies); (d) promoting engagement with influential actors; and (e) a process for goal-setting and feedback. The effect of interventions on network structures, or the moderating effect of structure on the intervention effectiveness was rarely assessed.

Conclusions: As many natural face-to-face opportunities for social connection are limited due to COVID19, groups already at risk for social isolation and loneliness are disproportionately impacted. Network-building interventions include multiple components that address both the structure of individuals' networks, and their skills and motivation for activating them. These intervention elements could be adapted for delivery via on-line platforms, and implemented by trained facilitators or novice volunteers, although more rigorous testing is needed.

Title: Individual- and Group-Level Network-Building Interventions to Address Social Isolation and Loneliness; A Scoping Review with implications for COVID19

INTRODUCTION

Exercising social/physical distancing and home quarantine is contributing to the increase of social isolation and loneliness [1], which may have a significant impact on the health and mental health of vulnerable populations [2]. Recent lockdowns due to COVID19 have brought the problems of social isolation and loneliness to the forefront of public attention [3], but these problems are not new. Loneliness is at least as old as the Epic of Gilgamesh [4] and there is evidence that both social isolation and loneliness have increased in the United States in recent years [5–8].

Loneliness and social isolation are complex, multilevel phenomena. Social isolation refers to the lack of social contacts and engagement [9], whereas loneliness reflects subjective dissatisfaction with the quality or quantity of social contacts [10]. They are independently associated with physical illness, mental illness, and mortality [11]. Everyone can experience varying intensity and duration of loneliness and/or isolation at a certain point in life [12]. However, some populations such as those who are older, people who are LGBT or who have cognitive disabilities, are disproportionately affected by social isolation and loneliness, while gender, health and income also play a role [13–15].

Loneliness/isolation intervention as network-building interventions

Interventions to address isolation/loneliness generally aim to improve the quantity and quality of social relations with existing or new support individuals and groups. Network-building interventions are deliberate efforts to change social networks of individuals [16]. Social network analysis (SNA) is an important approach to assess how interventions addressing loneliness/isolation change social networks. SNA is a well-established approach that focuses on the relational patterns between network actors [17, 18] rather than considering them as separate units. In other words, SNA captures the interdependencies among network actors, whereas conventional research methodologies assume independence among network participants [19].

A popular approach to study the network outcomes of loneliness/isolation interventions is assessing the change in the size of personal networks [20], or individuals' evaluation of support network [21, 22]. Many existing social network tools, including Berkman Syme Social Network Index [23] and Lubben Social Network Scale [24], ask about the number of individuals in each important social roles (family, friends, colleagues, etc), and usually provide a single scale. Some researchers ask about the number of important individuals in respondent's social network [25], create numerical scores based on a mix of the number and quality of social relations [26], or ask respondent's evaluation of the number of people who can help each other in the community [27].

In the following section, we argue that the number of ties does not fully capture the relational complexity of social networks in which one is embedded.

Network structure and well-being

Several aspects of network structure influence individuals' perception of loneliness and support. Here we provided a few examples:

Centrality represents the prominence of an actor in the network. The simplest measure of centrality, degree centrality, is the number of network connections an individual has (e.g. the number of friends, or family members) [28]. Degree centrality is one of the most commonly measured indicators of network structure, although authors do not necessarily refer to a count of connections as a measure of centrality [20]. In the case of social support, degree centrality could either be indegree (the number of people who offer you social support or who name you as a friend) or outdegree (the number of people to whom you offer social support or whom you name as a friend). Indegree centrality is positively correlated with emotional support [29]. However, a variety of other measures of centrality are associated with well-being. For example, betweenness centrality, the extent to which an individual is on paths between other members of the network, and closeness centrality, the extent to short paths connect the individual and other members of the network, are positively correlated with measures of wellness [30]. Indirect connections also matter. Being connected to more people indirectly (through others) decreases the likelihood of depression [31].

The **density** (connectedness) of networks affects well-being. Compared to degree centrality which is about the number of relations from or towards ego (respondent), density is about overall connectedness of personal network, which should include relationships among network members not including ego. Density of personal networks was shown to predict loneliness in students, particularly in men [32, 33]. However, some studies failed to show the association between density and subjective loneliness [34, 35], or proposed that the effect of density of personal networks on life satisfaction is moderated by individual's personality (as individuals may differ in terms of satisfaction by embedding in denser networks) [36].

Reciprocity (bi-directionality) of social relations matters for several reasons. Qualitative studies have found that older individuals have an easier time accepting social support when it is offered as part of a reciprocal relationship, that is, when they feel that they have given (in the case of their children) or are giving help to the person who is helping them [37, 38]. Individuals who perceive reciprocity in their relationship with their best friends feel less lonely [39]. There is experimental evidence that reciprocity is a key factor in building trust in networks [40]. Reciprocal imbalance in relationships (over-benefiting and under-benefiting) may lead to mental distress and less satisfaction with relations [41]. All of these findings suggest that reciprocity may be one key to maintaining relationships once they are established.

Network clustering, or the degree to which groups of three individuals connect completely with each other, is another important feature. In a longitudinal analysis of a large population-based study, Cacioppo and colleagues (2009) found that loneliness occurs in clusters, particularly clusters of individuals that are peripheral to the social network [43]. People in more clustered networks tend to be healthier [30]. Experiments find that the search for cooperative partners produces clustering, suggesting that clustered networks play a role in maintaining cooperation in groups [44, 45]. Of course, the influence that clustered networks have on their members has a downside. DiFonzo et al (2014) find that clustering increases the social influence of the group but also increases the tendency of groups to strongly differentiate themselves from others [47].

Based on the abovementioned evidence, we argue that interventions addressing loneliness/social isolation may affect the structure of social networks beyond merely increasing the number and frequency of social relations to ego. They may change the reciprocity of relations, may affect formation of denser clusters, or may even selectively affect some regions of one's social network (e.g. only improving the quality of intimate relations, or leading to bridging ties to new clusters). Given the need for more in depth analysis of the effect of interventions for loneliness/isolation, and lack of integration of social networks in interventions and outcomes measures, we conducted a systematic scoping review of studies that assessed how social network interventions that address loneliness and isolation affect the structure of social networks. We evaluated how these interventions can be adapted to promote connectedness in the wake of COVID19.

METHODS

We followed PRISMA-ScR guidelines for scoping reviews in literature review, data extraction/charting, and synthesis [48].

Literature Search

Our search strategy involved forward/backward citation tracking of three seminal network intervention publications, Valente (2012) [16], Valente et al. (2015) [49], and Latkin & Knowlton (2015) [50], complemented by a bibliographic search in Web of Science and SCOPUS, performed in October 2019 (search strategy in Online supplement). To capture articles about relevant interventions for social isolation and loneliness that might not have been described using network intervention key words, we also conducted a hand search of seminal reviews on the topic [20–22, 51]. Articles identified through this search strategy were imported into the web-based review program, Covidence [52], before undergoing title/abstract review.

Study Selection

To establish consistency in the study selection and data charting process, all authors completed a trial screening process in pairs and clarified points of conflict. We included studies that intentionally aimed to change aspects of social networks to address isolation/loneliness, measured the network structure as study outcomes and/or used them to inform interventional strategies, and were available in English. We excluded studies that only reported the number of individuals one is connected to (network size) or only provided aggregate measures of quality and quantity of relations (such as Berkman Syme Social Network Index and Lubben Social Network Scale).

Data Extraction and Synthesis

A data-charting form was jointly developed by the reviewers to determine variables to extract. Pairs of reviewers independently extracted a calibration set of studies. The reviewers regularly discussed the results and continuously updated the data-charting form. Synthesis of results was guided by the following three steps: 1) descriptive summary describing details about study design, 2) thematic analysis for the categorization of network interventions, and 3) consideration for how these interventions could be adapted to COVID19 restrictions.

RESULTS

Literature Search and Selection Process

Of our initial 3390 references, we assessed the full texts of 233 articles, of which 17 studies were about the effect of interventions on social networks to address loneliness and isolation, of which

9 studies were excluded, as they either only reported the number of individuals one is connected to or developed single aggregate measures for quality and quantity of relations without providing information about the composition of social network and/or detailed information about relations to particular social groups/roles. personal network size as a general indicator of social support. We included eight studies that measured network structure before and after the intervention and/or incorporated network analysis as a component of the intervention (Figure 1). Three studies assessed interventions focusing on *individual-level network building*, and four studies assessed *group-level network building interventions*. Kasari et al. (2016) compared an individual-level with a group-level intervention, and consequently was included in both sections [53].

Individual-Level Network Interventions
The interventions in this category generally aimed to help the individuals with limited social connections (e.g. older adults and children and adults with intellectual, mental, and physical disabilities) strengthen/activate existing social relations or engage in activities to facilitate new relations or strengthen current ties (Table 1).

Study Designs. Two studies were small case reports, small uncontrolled studies, or qualitative studies. Kasari et al. (2016) conducted an RCT comparing the individual-level intervention (matching subjects with typically developing peers in social activities and games) and a group-based social skills training (explained in the next section) [53]. Band et al. (2019) published the protocol for an upcoming pragmatic RCT of an online platform to develop personal maps, and connect adults to local and online activities and resources, compared to a wait-list control [54].

Individual-Level Network Intervention Components. Interventions usually included elements of motivational training, reflection, and goal setting.

The interventions in two studies included assisting the participants to develop personal network maps, and reflect on their structure. In Band et al. (2019)[54] and Osilla et al. (2016)[55], facilitators would help participants develop their personal maps, through an online platform in Band et al. (2019)[54], and a computer-assisted face-to-face discussion in Osilla et al. (2016)[55]. The online interface in Band et al. (2019)[54] provided concentric circles representing tie strength, and various social roles including individuals, groups, and pets. In Osilla et al. (2016)[55], the process included a structured network interview, followed by discussions on visualizations. In Osilla et al. (2016)[55], the participants also identified relationships between network members, also known as “alters” (whether alter 1 knows alter 2), but in Band et al. (2019)[54], the questions were all about the relationships with ego (the focal actor), with no mention of alter-to-alter relations.

In three studies, the intervention included facilitation of social contact and exposure, either through connection to local activities as a general opportunity for network building [53, 54, 56], or engaging in social activities with natural peers (or friends with no disabilities) [53]. In one study the intervention also included social skills training [56]. Two studies also included patients’ family or support staff in the intervention [55, 56]. All studies described interventions consisting of several sessions lasting multiple weeks, to provide enough time for participants to reflect on their networks and make changes over time.

Two studies examined social network interventions that were administered in-person. In Band et al. (2019)[54] the intervention is an online platform to develop personal maps, and connect adults to local and online activities and resources. Similarly, Osilla et al. (2016)[55] assessed the effect of a computer-assisted motivational network intervention, in which the process of data collection and presentation of network maps was done on a tablet.

Outcomes.
Two studies used sociometric network surveys to develop network structures and how they changed over time in students [53, 56]. Both studies calculated network salience/inclusion scores at each time point, using a method developed by Cairns & Cairns (1994)[57]. The score for each student is the ratio of the number of times they were identified by others belonging to a group to the largest baseline score in class at baseline. In their qualitative analysis, Osilla et al. (2016)[55] showed that visualization through the development of personal network maps was useful in helping participants build insight on the composition of their networks.

Group-Level Network Interventions
Interventions in this category were administered in small groups and, in addition to network-building at individual levels, also aimed to facilitate communication, support, and role-modeling among peers within the groups (Table 1).

Study Designs. Four studies were case reports and uncontrolled pre-post designs. Kasari et al. (2016)[53] conducted an RCT to compare an individual- and a group-level intervention.

Group-Level Network Intervention Components. Group-level interventions involved delivering social skills training [53, 58, 59], or providing opportunities for co-participation in activities of common interest [59]. Two studies involved peer support and training through pairing isolates with highly connected actors [60, 61], while another study encouraged peer support and training through co-participation of individuals and their support workers [58].

Three studies explicitly incorporated analyzing network structure into their interventions. In van Asselt-Goverts et al. (2018) [58], development of and reflection on personal network maps was a component of group training. Facilitators helped participants develop personal support maps using concentric circles representing tie strength, various social roles (e.g. family, friends, neighbors), and frequency, type, and preference of relations to each alter. However, no alter-to-alter relation data were collected. In two studies [60, 61], the focus and content of group activities were guided by *network* diagnostics that involved the structural analysis of social networks (such as identification of isolates and components/subgroups, and calculation of degree, density, reciprocity, transitivity, centralization, and average of inverse distance). The network diagnostics informed strategies to modify structural characteristics.

Outcomes. van Asselt-Goverts et al. (2018) [58] reported the size, frequency, and functional characteristics of social relations to important social roles (family, friends, colleagues, neighbors, others). Only Gesell et al. (2013)[60] studied change in network structure and descriptively reported the change in structural measures (e.g. density, reciprocity, clustering, isolates, etc.) over time. Kasari et al. (2016)[53] reported social network salience scores (see above). Tesdahl

(2015)[59] developed an exponential random graph model to assess how personal, interpersonal, and structural features of the network was associated with existence of ties between pairs of actor. The model showed that the total number of sessions that pairs of participants attended, as well as similarity in physical activity and pregnancy due date would significantly increase the chance that they are connected in the conversation network. However, they did not directly assess the effect of the intervention, since there were no parallel control group or longitudinal assessment of network formation.

DISCUSSION

In the face of COVID 19, it is critical to consider the factors that facilitate or impede the potential use of social network interventions to alleviate social isolation and loneliness. Our review identified multiple interventions that may mitigate social isolation and loneliness among vulnerable individuals and groups. We describe these programmatic elements, their implications for network interventions generally, and potential application to address social isolation and loneliness in the wake of COVID 19.

Networks as intervention components

In this review we identified key elements from both individual-level and group-level network-building interventions that address social isolation and loneliness in a framework shown in Figure 2. These elements, described below, consist of the following: (a) creating opportunities to build networks; (b) building social skills; (c) assessing network diagnostics; and (d) promoting network engagement.

Creating Opportunities for Networking. Both groups of studies (e.g., individual-level and group-level) included interventions that provided opportunities for socialization and networking via participation in events and activities of common interest [53, 54, 56]. When opportunities exist, either in-person or online, this strategy facilitates natural network-building and expansion of the boundaries of social networks to a larger population of individuals with common interests. However, it seems that mere provision of opportunities was ineffective in forming sustained social ties, and other active strategies are needed.

Building Social Skills. Building social skills through lectures, role modeling, and games were used in some individual- and group-level studies [53, 55, 56, 58, 59, 61]. This is particularly useful for individuals who need assistance in building and maintaining social relations. In some studies, the skillset trainings have been enhanced through peer learning and reflection [58, 59].

Network Diagnostics. Few studies formally used network structure in the intervention. Studies varied by using network maps as reflective tools and conversation facilitators, [54, 55, 58] to deliberate use of network analysis as a diagnostic tool [60, 61]. Few social network interventions in our study made any deliberate attempt to alter network structure. The GROW intervention, with its emphasis on connecting isolates to other group members and connecting clusters together is an exception [60, 61].

Promoting Network Engagement. Engagement of influential actors in the process of network-building was used in some studies. In individual-level interventions, this was accomplished by inclusion of significant network members (e.g. caregivers, immediate family) in the sessions [53,

56, 60, 61]; and in group-level interventions by matching novices with experts or isolates with central actors [60, 61]. Such strategies formally incorporate social influence and opinion-leadership in the network-building strategy.

Goal Setting, Feedback, and Update. Most of the included studies recognized the gradual process of network-building and the possibility of trial-and-error. Brief and short-term interactions are less likely to lead to sustainable network building. Consequently, incorporating this iterative and reflective process into interventions is an important consideration in designing network-building strategies that applies to all key elements introduced in Figure 2. Setting and updating goals based on feedback allows for individuals and groups to be actively engaged in network-building, leading to more promising network outcomes.

Since structural, social, interpersonal, and intrapersonal factors contribute to social isolation and loneliness [62], different components of network-building interventions (e.g. mapping, self-reflection, and creating networking opportunities) might be helpful within different contexts. Mapping, reflection, and maintenance of existing networks could be more helpful when existing ties have untapped potential. However, when existing networks are limited, overburdened, or lack the capacity to provide needed support, individuals might need to expand their networks by building ties with new contacts.

Networks as outcomes

Very few studies of interventions to address loneliness/isolation measure the structure of social networks. Even though included studies collected data on the structure of networks, their reported outcomes were mostly limited to basic measures, such as network size, or relative centrality of actors in the network. We did not find any study that assessed if and how interventions would affect the density, clustering, and reciprocity of relations, and whether their effect would focus on certain types of relations, or certain social circles (e.g. family, or intimate relations). Much of the literature assumes that the goal of intervention is to form links with individuals, who may then offer increased social support. This effectively (and sometimes explicitly) draws on a paradigm in which an individual's direct connections to friends and family bring various kinds of social support, most often emotional or instrumental [63]. This is somewhat disappointing, because social network structure matters beyond the simple number of connections.

There is evidence that network reciprocity encourages information diffusion [64]; and individuals find clustered networks, those with multiple closed triads, to be more supportive [65]. Clustered social networks, which include closed triads in which three people all know each other, are more supportive and more powerfully influence individuals than networks with more open triads [65, 66]. The quality of social network connections is also of concern [67]. Network structural and quality considerations could easily be implemented in a program such as Genie [54], and are quite amenable to online audio/video platforms such as Skype or Zoom.

Additionally, studies have not addressed the time over which network changes following intervention can be expected to endure. Interventions that strengthen relationships within existing subgroups, and provide support for their sustainment might be especially promising for reducing isolation.

Implications for Post-COVID19 Application

COVID19 pandemics and its lock-down and "social distancing" consequences worsened, and yet raised attention to the widespread problem of loneliness and its impacts on mental and physical health, particularly in older adults and vulnerable populations [68–72]. Many natural opportunities for face-to-face social communication and network-building will remain limited for the time being. Network-building interventions can potentially mitigate this problem in individuals most in need. Many of their key elements could be implemented online and the trainings and preparations do not seem to be heavily burdensome for health care systems. Our review can provide practical insights into the design and implementation of network-building interventions and potential considerations in adapting them to the needs of different target groups. In Figure 2, we present potential opportunities and barriers to implementation of each key element of network-building interventions.

Since the early stages of the COVID19 pandemic and in subsequent months, many health care systems adopted various tele-health care models [73, 74], so individuals connected to institutional or community services still, at least partially, benefit from their ties with professionals and staff virtually. In other words, social workers, home health aides, and other professionals connected to populations at risk of social isolation and loneliness may be well positioned to conduct network interventions. Their assistance is particularly valuable to people without natural connections within a household (e.g. widowed older adults). For instance, staff and volunteers in programs such as Meals on Wheels serving homebound older adults, are an obvious place to start. Consumers of such formal services could then suggest other peers who might benefit, leading to a snowball sample. Community-based programs have also been found to enhance the neighborhood social networks of older adults, potentially mitigating the risk for social isolation and loneliness [75]. Another possibility to expand social networks is to leverage the skills of paraprofessionals or even volunteers, rather than clinicians, as is done in self-help groups. While the successful Network Support program uses clinicians to facilitate the integration of recovering alcoholics into networks of sober peers [76], twelve step programs have accomplished the task for decades with and without professional assistance [77]. There are also programs that teach individuals' trusted natural ties, family and friends, to deliver social network interventions [26, 58]. The phrase, "Each one teach one," could easily be modified to, "Each one reach one."

Since structural, social, interpersonal, and intrapersonal factors contribute to social isolation and loneliness [62], different components of network-building interventions (e.g. mapping, self-reflection, and creating networking opportunities) might be helpful within different contexts. Mapping, reflection, and maintenance of existing networks could be more helpful when existing ties have untapped potential.

Scalability is an issue with a number of the social network interventions identified in our review. Not surprisingly, a number of the interventions were developed and tested using clinicians, who may be prohibitively expensive during a large-scale crisis. If scale is a challenge for interventions that focus on helping individuals to map and understand their networks, interventions that create groups for isolated individuals can forge connections without needing a facilitator. However, these interventions might be limited during social distancing. One possibility is to create groups online based on common interests. It might also be possible to

construct micro groups of three or four who would meet face to face either regularly or occasionally while practicing safe social distancing [59].

LIMITATIONS

We recognize that the studies included in this review were drawn from a larger study examining social network interventions across all levels (e.g., individuals, groups, communities, organizations). Thus, there may be additional strategies to support network building (e.g., interventions targeting larger communities and organizations) that were omitted from the present findings. Furthermore, due to the dearth of research that examine social network intervention outcomes, we focused on providing a picture of the typology and common elements of network-building interventions, rather than quality appraisal and synthesis of effectiveness, that could enhance the interpretability of our findings. The majority of these studies relied on uncontrolled research designs limiting our understanding of the effectiveness of these interventions, underscoring the need for more rigorous trials.

CONCLUSIONS

Network-building interventions to address social isolation/loneliness have different combinations of five key elements: (1) creating opportunities for networking and socialization, (2) building social skills, (3) informing the interventions by network analysis of personal and group networks, (4) engagement of influential network members, and (5) goals setting, feedback, and update. The choice of intervention elements is a decision that should be made in light of the nature of the social relations, characteristics of participants, expertise of the facilitators, and contextual factors (such as access to online communication resources, availability of local services, willingness and accessibility of network members). Little has been done to assess how network-building interventions actually change the structure of social networks, beyond simply the number of contacts. Future studies should focus on assessing the effect of intervention elements and their combinations, and the effect of interventions on network structural outcomes.

In the midst of COVID19 pandemic, we are in urgent need of innovative approaches for building and maintaining social networks among those at risk for social isolation and loneliness. Online interventions or a combination of online, phone, and in-person interventions may facilitate network building among vulnerable individuals and groups. Alternative forms of delivery (e.g. phone or mail) might be helpful for people with limited access to the internet. The main motivation to connect through group-level interventions could vary from commonalities in neighborhood, demographics, and common health conditions. Given the limited opportunities for group activities in online environments, specific attention should be paid to feasibility testing and adaptation. Attention should be paid to motivating and maintaining social engagement in the group context. Individual and group-level interventions should be delivered in stepwise, iterative, and reflective styles. More studies are needed to identify what combination of network-building elements works best under what conditions. Until very recently it seemed that there would be time to slowly build the evidence-base to address the increasing problem of loneliness. The current COVID19 pandemic will hopefully force researchers to speed up that time-table.

ACKNOWLEDGEMENTS

The authors thank Elena Navarro for her assistance in data screening and extraction.

CONFLICT OF INTEREST:

On behalf of all authors, the corresponding author states that there is no conflict of interest.

REFERENCES

1. Sood S (2020) Psychological effects of the Coronavirus disease-2019 pandemic. Res Humanit Med Educ 7:23–26
2. Vahia I, Blazer D, Smith G, et al (2020) COVID-19, Mental Health and Aging: A Need for New Knowledge to Bridge Science and Service. Am J Geriatr Psychiatry DOI: https://doi.org/10.1016/j.jagp.2020.03.007 sh
3. Halpert J (2020) How to manage your loneliness. New York Times 20–24
4. Mitchell S (2004) Gilgamesh: A new English version. Free Press, New York
5. Cigna (2020) Loneliness and the workplace: 2020 U.S report
6. Holt-Lunstad J (2017) The potential public health relevance of social isolation and loneliness: Prevalence, epidemiology and risk factors. Public Policy Aging Rep 27:127–130
7. National Academies of Science E and M (2020) Social Isolation and loneliness in older adults: Opportunities for the health care system. National Academies Press, Washington, D.C.
8. Pew Research Center (2009) Social isolation and new technology: How the internet and mobile phones impact Americans' social networks.
9. Berkman L (1983) The assessment of social networks and social support in the elderly. J Am Geriatr Soc 31:743–749
10. Ciolfi M, Jimenez F (2017) Social isolation and loneliness in older people: A closer look at definitions. In: Issues and Insights in Disability and Aging. University of Southern Maine, Muskie School of Public Service, Portland, ME.
11. Holt-Lunstad J, Smith T, Baker M, et al (2015) Loneliness and Social Isolation as Risk Factors for Mortality: A Meta-Analytic Review. Perspect Psychol Sci 10:227–237
12. De Jong Gierveld J, Van der Pas S, Keating N (2015) Loneliness of Older Immigrant Groups in Canada: Effects of Ethnic-Cultural Background. J Cross Cult Gerontol 30:251–268
13. Anderson G, Thayer C (2018) Loneliness and social connections: A national survey of adults 45 and older. Washington. AARP Research, Washington, DC
14. Nicholson N (2012) A Review of Social Isolation: An Important but Underassessed Condition in Older Adults. J Prim Prev 33:137–152
15. Ong A, Uchino B, E W (2016) Loneliness and health in older adults: A mini-review and synthesis. Gerontology, 62:443–449
16. Valente T (2012) Network Interventions. Science (80-) 337:49–53
17. Cross R, Parker A, Borgatti S (2002) Making invisible work visible: Using social network analysis to support strategic collaboration. Calif Manage Rev 44:25-46
18. Fattore G, Frosini F, Salvatore D, Tozzi V (2009) Social network analysis in primary care: the impact of interactions on prescribing behaviour. Health Policy (New York) 92:141–8
19. Cranmer S, Desmarais B, Morgan J (2020) Inferential Network Analysis (Analytical Methods for Social Research). Cambridge University Press
20. Anderson K, Laxhman N, Priebe S (2015) Can mental health interventions change social networks? A systematic review. BMC Psychiatry 15:297
21. Webber M, Fendt-Newlin M (2017) A review of social participation interventions for people with mental health problems. Soc Psychiatry Psychiatr Epidemiol 52:369–380
22. Ma R, Mann F, Wang J, et al (2019) The effectiveness of interventions for reducing

subjective and objective social isolation among people with mental health problems: a systematic review. Soc Psychiatry Psychiatr Epidemiol https://doi.org/10.1007/s00127-019-01800-z
23. Berkman L, Syme S (1979) Social networks, host resistance, and mortality: a nine-year follow-up study of Alameda County residents. Am J Epidemiol 109:86–204
24. Lubben J (1988) Assessing social networks among elderly populations. Fam Community Health 11:42–52
25. Fyrand L, Moum T, Finset A, Glennås A (2003) The effect of social network intervention for women with rheumatoid arthritis. Fam Process 42:71–89. https://doi.org/10.1111/j.1545-5300.2003.00071.x
26. Terzian E, Tognoni G, Bracco R, et al (2013) Social network intervention in patients with schizophrenia and marked social withdrawal: a randomized controlled study. Can J Psychiatry 58:622–631
27. Harada K, Masumoto K, Katagiri K, et al (2018) Community intervention to increase neighborhood social network among Japanese older adults. Geriatr Gerontol Int 18:462–469
28. Marsden P (2002) Egocentric and sociocentric measures of network centrality. Soc Networks 24:407–422
29. Lakon C, Hipp J, Timberlake D (2010) The Social Context of Adolescent Smoking: A Systems Perspective. Am J Public Health 100:1218–1228
30. Lin S, Faust L, Robles-Granda P, et al (2019) Social network structure is predictive of health and wellness. PLoS One 14:e0217264
31. Rosenquist J, Fowler J, Christakis N (2011) Social network determinants of depression. Mol Psychiatry 16:273–281
32. Stokes J, Levin I (1986) Gender differences in predicting loneliness from social network characteristics. J Pers Soc Psychol 51:1069–1074
33. Berg JH, McQuinn RD (1989) Loneliness and aspects of social support networks. J Soc Pers Relat 6:359–372
34. Green L, Richardson D, Lago T, Schatten-Jones E (2001) Network correlates of social and emotional loneliness in young and older adults. Personal Soc Psychol Bull 27:281–288
35. Brown R, Roberts S, Pollet T (2018) Loneliness is negatively related to Facebook network size, but not related to Facebook network structure. PsyArXiv https://doi.org/10.31234/osf.io/s2kya
36. Zou X, Ingram P, Higgins E (2015) Social networks and life satisfaction: The interplay of network density and regulatory focus. Motiv Emot 39:693–713
37. Breheny M, Stephens C (2009) "I sort of pay back in my own little way": managing independence and social connectedness through reciprocity. Ageing Soc 29:1295–1313
38. Boneham M, Sixsmith J (2006) The voices of older women in a disadvantaged community: issues of health and social capital. Soc Sci Med 62:269-279.
39. Buunk B, Prins K (1998) Loneliness, exchange orientation, and reciprocity in friendships. Pers Relatsh 5:1–14
40. Molm L, Whitham M, Melamed D (2012) Forms of Exchange and Integrative Bonds: Effects of History and Embeddedness. Am Sociol Rev 77:141–165
41. Fyrand L (2010) Reciprocity: A predictor of mental health and continuity in elderly people's relationships? A review. Curr Gerontol Geriatr Res 340161, https://doi.org/10.1155/2010/340161

42. Wellman B, Frank K (2001) Network capital in a multi-level world: Getting support from personal communities. In: Lin N, Burt R, Cook K (eds) Social capital: Theory and research. Aldine de Gruyter, Chicago, pp 233–274
43. Cacioppo J, Fowler J, Christakis N (2009) Alone in the Crowd: The Structure and Spread of Loneliness in a Large Social Network. J Personal Soc Psychol 97:977–991
44. Fehl K, van der Post D, Semmann D (2014) Co-evolution of behavior and social network structure promotes human cooperation. Ecol Lett 14:546–551
45. Melamed D, Harrell A, Simpson B (2018) Cooperation, clustering, and assortative mixing in dynamic networks. Proc Natl Acad Sci 115:951–956
46. Tracy E, Min M, Park H, et al (2016) Personal network structure and substance use in women by 12 months post treatment intake. J Subst Abuse Treat 62:55–61
47. DiFonzo N, Suls J, Beckstead J, et al (2014) Network Structure Moderates Intergroup Differentiation of Stereotyped Rumors. Soc Cogn 32:409–448
48. Tricco A, Lillie E, Zarin W, et al (2018) PRISMA extension for scoping reviews (PRISMA-ScR): checklist and explanation. Ann Intern Med 169:467–473
49. Valente TW, Palinkas LA, Czaja S, et al (2015) Social network analysis for program implementation. PLoS One 10:e0131712
50. Latkin CA, Knowlton AR (2015) Social Network Assessments and Interventions for Health Behavior Change: A Critical Review. Behav Med 41:90–97
51. Mann F, Bone J, Lloyd-Evans B, et al (2017) A life less lonely: the state of the art in interventions to reduce loneliness in people with mental health problems. Soc Psychiatry Psychiatr Epidemiol 52:627–638
52. Veritas Health Innovation (2019) Covidence systematic review software
53. Kasari C, Dean M, Kretzmann M, et al (2016) Children with autism spectrum disorder and social skills groups at school: A randomized trial comparing intervention approach and peer composition. J Child Psychol Psychiatry 57:171–179
54. Band R, Ewings S, Cheetham-Blake T, et al (2019) Study protocol for 'The Project About Loneliness and Social networks (PALS)': a pragmatic, randomised trial comparing a facilitated social network intervention (Genie) with a wait-list control for lonely and socially isolated people. BMJ Open 9:e028718
55. Osilla KC, Kennedy DP, Hunter SB, Maksabedian E (2016) Feasibility of a computer-assisted social network motivational interviewing intervention for substance use and HIV risk behaviors for housing first residents. Addict Sci Clin Pract 11:14
56. Locke J, Kang- Yi C, Pellecchia M, et al (2019) It's messy but real: a pilot study of the implementation of a social engagement intervention for children with autism in schools. J Res Spec Educ Needs 19:135–144. https://doi.org/10.1111/1471-3802.12436
57. Cairns R, Cairns B (1994) Lifelines and risks: Pathways of youth in our time. Cambridge University Press, New York
58. van Asselt-Goverts AE, Embregts PJCM, Hendriks AHC, et al (2018) Evaluation of a social network intervention for people with mild to borderline intellectual disabilities. J Appl Res Intellect Disabil 31:e229–e243. https://doi.org/10.1111/jar.12318
59. Tesdahl E, Gesell SB (2015) Assessing the Impact of De Novo Social Ties within Health Intervention Settings: New Questions for Health Behavior Intervention Research. Cts-Clinical Transl Sci 8:676–681
60. Gesell SB, Barkin SL, Valente TTW (2013) Social network diagnostics: a tool for monitoring group interventions. Implement Sci 8:116

61. Gesell SB, Barkin SL, Sommer EC, et al (2016) Increases in Network Ties Are Associated With Increased Cohesion Among Intervention Participants. Heal Educ Behav 43:208–216
62. Courtin E, Knapp M (2017) Social isolation, loneliness and health in old age: a scoping review. Health Soc Care Community 25:799- 812
63. Gariépy G, Honkaniemi H, Quesnel-Vallée A (2016) Social support and protection from depression: Systematic review of current findings in Western countries. Br J Psychiatry 209:284–293
64. Zhu Y, Zhang X, Sun G, et al (2014) Influence of Reciprocal Links in Social Networks. PLoS One 9:e103007
65. Lee S, Chung J, Park N (2016) Linking cultural capital with subjective well-being and social support: The role of communication networks. Soc Sci Comput Rev 34:172–196
66. Malik N, Shi F, Lee H, Mucha P (2016) Transitivity reinforcement in the coevolving voter model. Chaos An Interdiscip J Nonlinear Sci 26:123112
67. Cacioppo S, Grippo A, London S, et al (2015) Loneliness: Clinical import and interventions. Perspect Psychol Sci 10:238–249
68. Armitage R, Nellums L (2020) COVID-19 and the consequences of isolating the elderly. Lancet Public Heal 5:e256
69. Courtet P, Olié E, Debien C, Vaiva G (2020) Keep socially (but not physically) connected and carry on: preventing suicide in the age of COVID-19. J Clin Psychiatry 81:e20com13370
70. Banerjee D, Rai M (2020) Social isolation in Covid-19: The impact of loneliness. Int J Soc Psychiatry doi.org/10.1177/0020764020922269
71. Okruszek L, Aniszewska-Stańczuk A, Piejka A, et al (2020) Safe but lonely? Loneliness, mental health symptoms and COVID-19. https://doi.org/1031234/osf.io/9njps
72. Marziali M, Card K, McLinden T, et al (2020) Physical distancing in COVID-19 may exacerbate experiences of social isolation among people living with HIV. AIDS Behav doi.org/10.1007/s10461-020-02872-8
73. Smith A, Thomas E, Snoswell C, et al (2020) Telehealth for global emergencies: Implications for coronavirus disease 2019 (COVID-19). J Telemed Telecare doi.org/10.1177/1357633X20916567
74. Hollander J, Carr B (2020) Virtually perfect? Telemedicine for COVID-19. N Engl J Med 382:1679–1681
75. Harada K, Masumoto K, Katagiri K, et al (2018) Community intervention to increase neighborhood social network among Japanese older adults. Geriatr Gerontol Int 18:462–469. https://doi.org/10.1111/ggi.13208
76. Litt M, Kadden R, Tennen H, Kabela-Cormier E (2016) Network Support II: Randomized controlled trial of Network Support treatment and cognitive behavioral therapy for alcohol use disorder. Drug Alcohol Depend 165:203–212
77. Kelly J, Humphreys K, Ferri M (2020) Alcoholics Anonymous and other 12- step programs for alcohol use disorder. Cochrane Database Syst Rev Art. No.: CD012880.
78. Chan A, Yu D, Choi K, Chan D. S. F.;Choi, K. C. AWK ;Y. (2017) Effects of tai chi qigong on psychosocial well-being among hidden elderly, using elderly neighborhood volunteer approach: a pilot randomized controlled trial. Clin Interv Aging 12:85–96. https://doi.org/10.2147/cia.S124604

Figure 1: PRISMA Flow Diagram of the study selection

Figure 2: Components of individual- and group-level network-building interventions; opportunities and barriers for post-covid19 adaptation

Table 1: Study characteristics, elements of network-building interventions, and main outcome measures

Study	Study design	Setting	Network actors	Network ties	Network-building intervention	Network Analysis procedures and outcomes	Other measures of effectiveness
**Individual-level interventions**							
Band et al. (2019)	Pragmatic RCT with a wait-list control	Community organizations in two areas in the UK.	Adults who are isolated or at risk of loneliness; n=394	Ties with family members, friends, acquaintances, healthcare professionals, local groups and pets	Generating Engagement in Network Involvement (GENIE): Online social networking tool to create opportunities for social involvement through social network mapping, tailoring of preferences and linking users to valued resources and activities, delivered by trained facilitators. Facilitators helped participants develop personal support maps using concentric circles representing tie strength, and various social roles including individuals, groups, and pets [no mention of alter-to-alter relations]	Social network composition change (only in intervention arm) [since no alter-to-alter relations are collected, the composition will likely only refer to ego's relations]	Study in-progress<br>SF-12 Mental Health and Physical Health Composite scores<br>Loneliness, social isolation, well-being, Quality-adjusted life years, cost, Capability well-being, Collective efficacy in Network scale, engagement in new activities
Locke, et al. (2019)	Case report, feasibility pilot	Elementary school	Elementary school students with Autism Spectrum Disorders; n=4	Observed playground social interactions & in-class friendship ties	Remaking Recess is an adult-facilitated intervention to support social engagement of children with ASD when at recess. The intervention includes observing if children need additional support to engage with their peers, follow children's lead, strengths and interests, providing direct instruction on social skills and games and activities to scaffold engagement, creating opportunities for reciprocal interactions, sustain engagement within an activity, coaching through difficult peer situations, work with typically developing peers to engage children with ASD, and eventually fading out supports to promote independence.	Sociometric survey of friendship networks within each class about students' social network and their perception of "who hang out together". Centrality of students at class (number of times the student identified by others as belonging to a group) was used to calculate social network inclusion score (individual's centrality at each time divided by highest baseline centrality in class).<br><br>Social network inclusion scores initially increased but declined at 6 week follow-up.	Decrease in solitary playground time; increase in joint engagement with peers.

Osilla et al. (2016)	Qualitative feasibility and acceptability assessment	Organization that provides permanent supportive housing	Formerly homeless people with histories of AOD and HIV risk behaviors; n=11	Social interactions	Computer-assisted Motivational Network Intervention: Across 4 sessions (2-week intervals), participants complete a survey on drinking and unprotected sex and a name generator network survey followed by motivational discussions on the network map, the pros and cons of the patterns in the map, opportunities to discuss positive behavior change and goal setting for the following week.	Residents reported that the intervention was helpful in discussing their social network, that seeing the network maps was more impactful than just talking about their network, and that the intervention prompted thoughts about changing their AOD use and HIV risk networks.	
Kasari et al. (2016)	RCT, comparing SKILLS (group social skill training) and ENGAGE (peer-matched social activities)	Elementary schools	Children with Autism Spectrum Disorder; n=148	Friendship ties	SKILLS: 16 sessions of group participation in interactive classes on social skills training: Being a Social Detective; Greetings and Goodbyes; Body Talk (Nonverbals); Humor; Conversation; Dealing with Teasing; Perspective Taking; Dealing with Emotions; and Friendship Tips.<br><br>ENGAGE: Children with ASD and their typically developing peers (nominated through friendship survey or teacher nomination) engage in 16 sessions of group play activities, collectively establishing a daily schedule in order to encourage cohesiveness, conversational exercises, structured games, free play, improvised storytelling, and music. Peers were encouraged to take leadership of their own groups with supervision from adults as needed.	Sociometric survey of friendship networks within each class about students' social network and their perception of "who hang out together". Centrality of students at class (number of times the student identified by others as belonging to a group) was used to calculate social network Salience score (individual's centrality at each time divided by highest baseline centrality in class).<br><br>No significant change in network salience scores over time by study group.	More playground engagement for SKILLS group than for ENGAGE group; All children significantly increased percentage of time spent engaged with peers.
**Group-level interventions**							
van Asselt-Goverts, et al. (2018)	Case report, qualitative assessment	Organization providing support for people with intellectual disabilities in Netherlands	Adults with mild to borderline intellectual disabilities and their support workers; n=5	Ties to family, friends, neighbors, colleagues, acquaintances & professionals	A 7-session semi-structured group training to strengthen or expand the networks of participants. Sessions were facilitated by experienced trainers and focused on talents and interests, network, neighborhood, wishes and dreams, plans for a supporters meeting, and evaluation. Exercises included role playing and making a personal map. Facilitators helped participants develop personal support maps using concentric circles	Increased network size, frequency, and quality of relationships. The results were presented for individual participants and varied by individuals. No structural measures of networks were reported.	Decreased loneliness, increased awareness, competence, autonomy and increased participation.

					representing tie strength, and various social roles including family, friends, neighbors, colleagues, other acquaintances, and professionals [no mention of alter-to-alter relations]. For each network member, the participants scored the frequency of contact, affection, types of support, and preferences. Participants re-evaluated their maps after the intervention.		
Gesell et al. (2013)	Uncontrolled before-after	Community recreation center in Nashville, TN	Parents of children at risk for obesity; n=11	Advice and discussion ties with group members outside of training sessions	GROW intervention: Participants responded to online surveys at week 4 and 12, identified their advice and discussion partners from the list of cohort members. Network diagnostic tool involved identification of isolates and components/subgroups, and calculation of degree, density, reciprocity, transitivity, centralization, and average of inverse distance.<br><br>The network-building intervention involved 12-week group skill building sessions in which social network diagnostics were used to create an action plan and recommendations for each group and its leader and a menu of action steps. Group sessions functioned to establish a strong group identity through developing and working toward a shared common goal and group identity. Group social networks were restructured through strategic pairing of isolates with highly connected group members, calling isolates in groups and promoting their participation, pairing non-reciprocated links, bringing triads together, and pairing members from different subgroups in small group activities; re-assigning members to prevent formation of silos.	Number of advice and discussion partners increased over time (non-significant); Advice network density increased significantly; Number of isolates remained stable; Subgroups decreased (integrated into network); Centralization increased in advice network and decreased in discussion network;	No change in perceived cohesion.
Gesell et al. (2016)	Uncontrolled before-after	Community recreation center in Nashville, TN	Parents of children (3-6 years old) at risk for obesity; n=305	Advice and discussion ties with group members outside of training	GROW intervention; explained above	Significant increase (from week 3 to 6) in advice nominations; Non-significant increase in discussion nominations;	Significant increase (from week 3 to 6) in cohesion; Non-significant increase in discussion nominations; New network nominations were associated with perceptions of

				sessions			group cohesion.
Tesdahl et al. (2015)	Uncontrolled before-after	Community recreation center in Nashville, TN	Expectant mothers; n=59	People within the program with whom the respondent spoke about pregnancy-related health issues	The intervention included small-group activities aimed at achieving a common goal (e.g., planning an event for family and friends) with rotating leadership roles to increase group cohesion. The intervention also included social skills practice for building and strengthening positive support among family and friends, including identifying existing supports for prenatal health as well as gaps in support networks, identifying the benefits and attributes of supportive relationships, and learning how to build new and tend to supportive relationships.	Participants responded to name generator surveys at weeks 6 and 12 (later mixed together to create cross-sectional network) to identify other study participants with whom they have spoken about well-being.<br><br>Participants created 3.5 ties on average. Only %4 ties at week 6 remained the same at week 12. The merged cross-sectional network of weeks 6 and 12 was analyzed using Exponential Random Graph Modeling, with tie existence between pairs of participants as dependent variable, and total number of sessions the pair attended as the independent variable indicating program effect.<br><br>Attendance significantly affected the formation of network ties among pairs of participants. Similarity in physical activity level and due dates increased the likelihood of tie formation.	

Figure 1

Click here to access/download;Figure;Figure

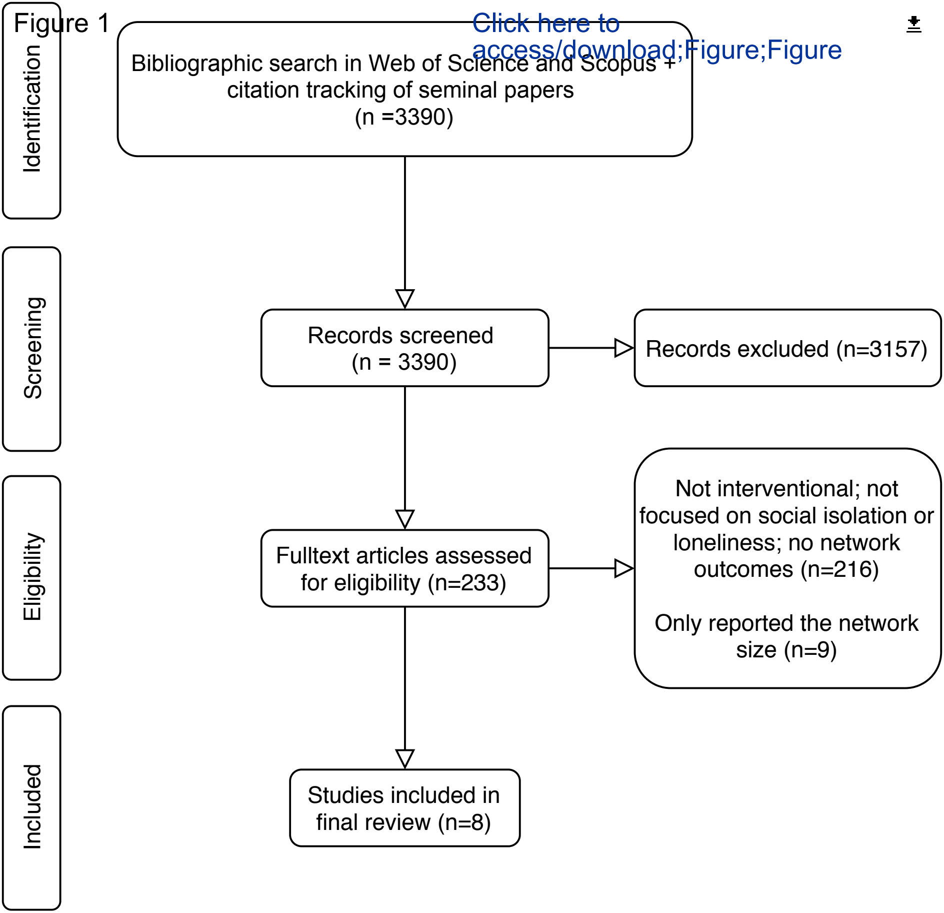

Figure 2

Click here to access/download;Figure;Figure 2.pdf

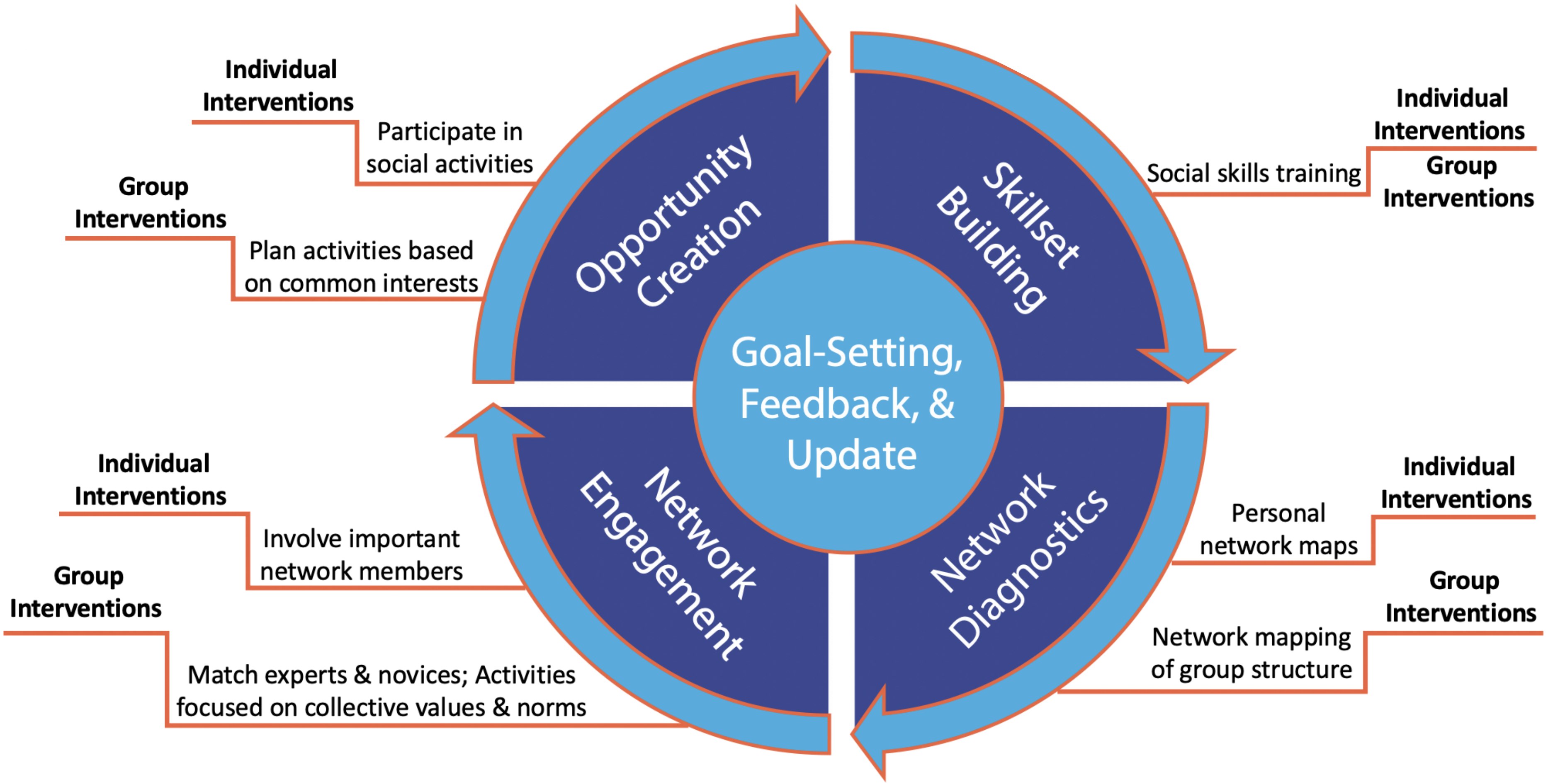

Click here to access/download
Supporting Information
Online supplement.docx
